# Preoperative Assessment Using CT and MRI Scans of the Temporal Bone to Determine the Degree of Difficulty in Cochlear Implant Surgery

**DOI:** 10.7759/cureus.65196

**Published:** 2024-07-23

**Authors:** Stany Jerosha, Sakthi Ganesh Subramonian, Arunkumar Mohanakrishnan, Yuvaraj Muralidharan, Paarthipan Natarajan

**Affiliations:** 1 Radiodiagnosis, Saveetha Medical College and Hospital, Saveetha Institute of Medical and Technical Sciences, Saveetha University, Chennai, IND

**Keywords:** surgical difficulty prediction, hrct and mri, preoperative assessment, temporal bone imaging, cochlear implant (ci) surgery

## Abstract

Background

Cochlear implant surgery is a complex procedure influenced by the anatomical structures of the temporal bone. Preoperative imaging using CT and MRI can provide critical insights into the surgical challenges that may be encountered. This study aims to evaluate the role of CT and MRI in preoperative assessment to predict the difficulty of cochlear implant surgery in terms of surgical time.

Materials and methods

A retrospective observational study was conducted at Saveetha Medical College and Hospital, Chennai, from April 2022 to September 2023. Ninety patients with severe to profound sensorineural hearing loss who underwent cochlear implantation were included. Preoperative high-resolution CT (HRCT) and MRI of the temporal bone were performed to assess various anatomical parameters. Surgical difficulty was evaluated intraoperatively and correlated with preoperative imaging findings. Data were analyzed using IBM SPSS Statistics for Windows, V. 21.0 (IBM Corp., Armonk, NY).

Results

The mean age of participants was 7.4±10.9 years, with the majority (66.7%) in the 1-5-year age group. Out of 90 participants, 50 were male and 40 were female. HRCT and MRI revealed that 35.6% of participants had hypo-/non-pneumatized mastoids, 3.3% had narrow facial recesses, and 3.3% had high-riding jugular bulbs. Significant correlations were found between surgical time and associated congenital (p=0.006) and acquired (p=0.0001) anomalies of the temporal bone, as well as the total difficulty score (p=0.0001). The mean surgical time was 103.97±25.2 minutes, with a range from 45 to 220 minutes.

Conclusion

Preoperative HRCT and MRI are valuable tools in predicting the degree of difficulty in cochlear implant surgery. Specific anatomical features identified in imaging studies can significantly influence the surgical approach and duration. These findings underscore the importance of detailed preoperative imaging to enhance surgical planning and outcomes in cochlear implant procedures.

## Introduction

Cochlear implants represent a revolutionary advancement in treating severe to profound sensorineural hearing loss. Unlike hearing aids that amplify sound, cochlear implants convert sound energy into electrical impulses that directly stimulate the auditory nerve, bypassing damaged hair cells in the inner ear. This transformative technology is particularly beneficial for individuals who do not experience significant improvement with traditional hearing aids [1].

Hearing impairment affects over 5% of the global population, with approximately 360 million people suffering from disabling hearing loss. This condition is most prevalent in low- and middle-income countries [2]. Among the hearing-impaired population, about 10% have severe to profound hearing loss, significantly impacting their quality of life and communication abilities [3]. The World Health Organization (WHO) estimates that 32 million children are among those affected, highlighting the critical need for effective interventions like cochlear implants [4].

The success of cochlear implantation largely depends on the anatomical and pathological conditions of the temporal bone. Accurate preoperative assessment using high-resolution CT (HRCT) and MRI of the temporal bone is essential to identify potential challenges and plan the surgical approach. These imaging modalities provide detailed insights into the structures of the inner ear, the mastoid bone, and surrounding critical anatomical landmarks, which are crucial for determining the feasibility and complexity of the surgery [5].

Previous studies have underscored the importance of preoperative imaging in predicting surgical outcomes and potential complications. For instance, anatomical variations such as a hypo-/non-pneumatized mastoid, narrow facial recess, and high-riding jugular bulb can significantly increase the complexity of cochlear implant surgery. Recognizing these variations preoperatively allows surgeons to anticipate difficulties and modify their surgical techniques accordingly, potentially reducing the risk of complications and improving overall surgical outcomes [6].

This study aims to evaluate the role of HRCT and MRI in the preoperative assessment of the temporal bone to predict the degree of difficulty in cochlear implant surgery. By correlating preoperative imaging findings with intraoperative challenges and surgical times, this research seeks to enhance the understanding of how detailed imaging assessments can contribute to better surgical planning and patient outcomes in cochlear implantation [7].

Understanding the intricate details provided by HRCT and MRI can aid in anticipating surgical challenges, thereby improving the precision and safety of cochlear implant procedures. This research will contribute valuable insights into the optimization of preoperative planning and the overall success rates of cochlear implant surgeries.

## Materials and methods

This study, conducted at Saveetha Medical College and Hospital in Chennai, included patients visiting the Department of Radiology as either in-patients or out-patients. It employed a retrospective observational design from April 2022 to September 2023. The study population encompassed patients of all age groups and both sexes who were candidates for cochlear implant surgery and were referred for further diagnostic work-up. If a patient underwent simultaneous bilateral cochlear implant surgery, it was counted as two subjects. Inclusion criteria were patients with bilateral severe to profound sensorineural hearing loss who had been assessed by CT and MRI and subsequently undergone cochlear implantation. Exclusion criteria included active middle ear disease, congenital aural dysplasia, medically unfit patients, and those unwilling to give informed consent.

The study aimed to compare the sensitivity of CT and MRI of the temporal bone in determining the difficulty of cochlear surgery. The sample size, calculated based on a correlation of 0.892 with surgical time as reported by Vaid et al., required 89 subjects to estimate with a margin of error of 0.4 and a 95% confidence level. The sample size is calculated using the formula ​n=Z^2^ P (1-P)/d^2^. In this formula, n represents the required sample size. The term Z is the Z-value (Z-score) corresponding to the desired confidence level, such as 1.96 for a 95% confidence level. The variable P denotes the estimated proportion of the population with the characteristic of interest, while (1−P) represents the proportion of the population without the characteristic (the complement of P). Finally, d stands for the desired margin of error or the precision of the estimate. 

For the study, the ethical committee granted a waiver of consent to access patient data from hospital records. Both scientific and ethical committee clearance was secured for the study. Data collection involved enrolling every subsequent patient presenting to the ENT OPD with a clinical history suggestive of bilateral sensorineural hearing loss and meeting the inclusion criteria. After proper counseling and informed consent, these patients underwent a detailed examination process.

The examination process included obtaining a comprehensive history, covering prenatal, perinatal, and postnatal birth history, family history of hearing loss, developmental milestones, speech and hearing evaluation, age of onset, immunization, socioeconomic history, and psychological evaluation. Routine ENT examinations included a complete ear examination, nose examination to assess the airway and nasopharynx, and throat examination of the oral cavity and oropharynx. Additionally, a routine CNS examination was conducted. Preoperative audiological testing involved behavioral audiometry, brainstem evoked response audiometry (BERA), otoacoustic emissions (OAE), auditory steady-state response (ASSR), tympanometry, and audiometry to assess the degree and type of hearing loss.

Preoperative imaging involved an MRI of the posterior fossa to evaluate the cochleovestibular nerve on both sides and HRCT and MRI scans of the temporal bone to examine typical cochlear anatomy and any existing diseases. These imaging studies were performed under the supervision of a senior radiologist experienced in temporal bone imaging. The HRCT scans were conducted using a Siemens SOMATOM Definition Edge 128-slice dual-energy machine (Siemens, Munich, Germany) with parameters including 128×0.625 collimation, 0.67 mm slice thickness, 0.33 mm increment, 360 reconstruction algorithm, 0.5 s rotation time, 0.426 pitch factor, and a 768×768 image display matrix. MRI was performed on a 1.5 Tesla GE Discovery 750 MRI machine (GE HealthCare, Chicago, IL) using a neurovascular or head coil, following a protocol that included sequences like diffusion, T2 axial, T2 fluid-attenuated inversion recovery (FLAIR) axial, T1 coronal, T2 coronal, T1 sagittal, axial T2 fast imaging employing steady-state acquisition (FIESTA) through the inner ear with 0.4 mm slices, and an oblique T2 balanced fast field-echo (BFFE) sequence.

All HRCT and MRI scans were examined in axial planes parallel to the long axis of the lateral semicircular canals, and coronal sections were viewed perpendicular to the axial plane. An expert in head and neck imaging evaluated each preoperative imaging modality. The data collected was entered into Microsoft Excel and analyzed using IBM SPSS Statistics for Windows, V. 21.0 (IBM Corp., Armonk, NY). Univariate and bivariate analyses were performed, with descriptive statistics used to calculate frequencies of categorical variables and measures of central tendencies and dispersion for continuous variables. The Pearson correlation coefficient was used to assess the correlation between surgical time and different scores, with a p-value of <0.05 considered statistically significant.

Ethical considerations were rigorously adhered to. Written and informed consent was secured from all participants prior to the study. Participants were assured of the complete confidentiality of their information and were given the option to withdraw from the study at any point. The study's methodology ensured no risk to the subjects, their family members, or the investigator, maintaining the highest ethical standards throughout the research process.

## Results

Demographic data

The study included 90 participants, with a mean age of 7.4±10.9 years. The age distribution ranged from one to 64 years, with the median age being four years. The majority of participants (66.7%) were in the 1-5-year age group, followed by 20% in the >5-10-year age group, 6.7% in the >10-18-year age group, and another 6.7% in the >18-year age group. The distribution of participants according to age group is shown below (Table 1).

**Table 1:** Distribution of participants according to age group

Age group	Frequency	Percent
1-5 years	60	66.7
>5-10 years	18	20.0
>10-18 years	6	6.7
>18 years	6	6.7
Total	90	100.0

Gender distribution

Out of the 90 participants, 50 (55.6%) were male and 40 (44.4%) were female.

Laterality of surgery

Of the 90 cochlear implant surgeries performed, 74 (82.2%) were on the right side and 16 (17.8%) were on the left side.

Radiological findings

The preoperative HRCT and MRI scans provided detailed insights into the anatomical variations and potential challenges for each surgery.

Mastoid pneumatization

**Figure 1:**
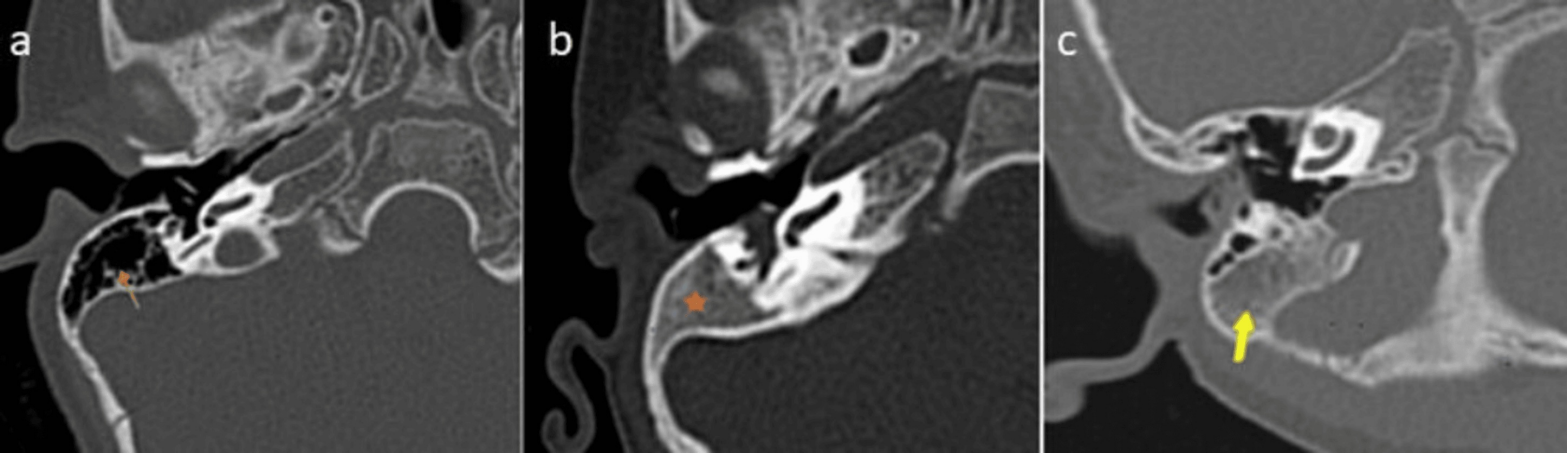
(a-c) Mastoid pneumatization HRCT of the temporal bone showing (a) a well-pneumatized mastoid (arrow), (b) a non-pneumatized right mastoid (star), and (c) few mastoid air cells indicating a hypopneumatized mastoid. HRCT: high-resolution CT

In the study, 58 participants (64.4%) exhibited well-pneumatized mastoids, while 32 participants (35.6%) had hypo- or non-pneumatized mastoids. Various types of mastoid pneumatization were observed as shown in Figure 1.

Facial recess anatomy

**Figure 2:**
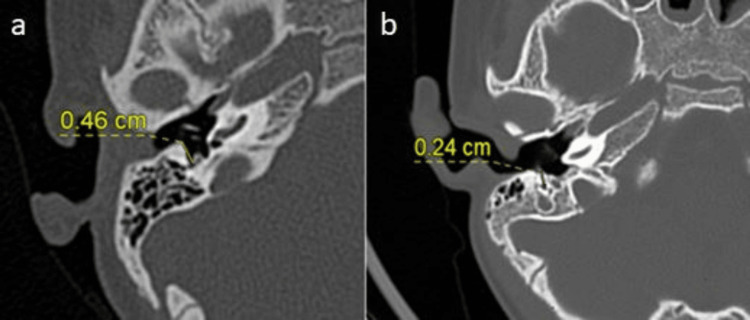
(a,b) Facial recess types HRCT of the temporal bone showing (a) wide facial recess (>3 mm) and (b) narrow facial recess (<3 mm). HRCT: high-resolution CT

Among the participants, 87 (96.7%) had wide facial recesses (>3 mm), while three (3.3%) had narrow facial recesses (<3 mm). Both wide and narrow facial recesses were documented, as shown in Figure 2.

Descending segment of the facial canal

All 90 participants (100%) exhibited normal findings.

Position of the jugular bulb

**Figure 3:**
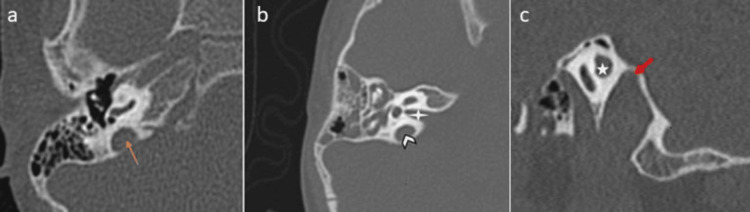
(a-b) Position of jugular bulb types (a) HRCT of the temporal bone showing the normal position of the jugular bulb (arrow), (b) axial image of HRCT of the temporal bone showing the superior part of the jugular bulb (arrowhead) seen at the level above the floor of the internal auditory canal (star), considered as a high-riding jugular bulb, and (c) reformatted sagittal plane of HRCT of the temporal bone showing the superior margin (red arrow) of the jugular bulb extending above the floor of the ipsilateral internal auditory canal (star). HRCT: high-resolution CT

Among the participants, 87 (96.7%) had normal jugular bulb positions, while three (3.3%) had high-riding or dehiscent jugular bulbs. Both normal and high-riding jugular bulb positions were noted, as shown in Figure 3.

Posterior wall of external auditory canal (EAC)/sigmoid sinus (SS) lines

**Figure 4:**
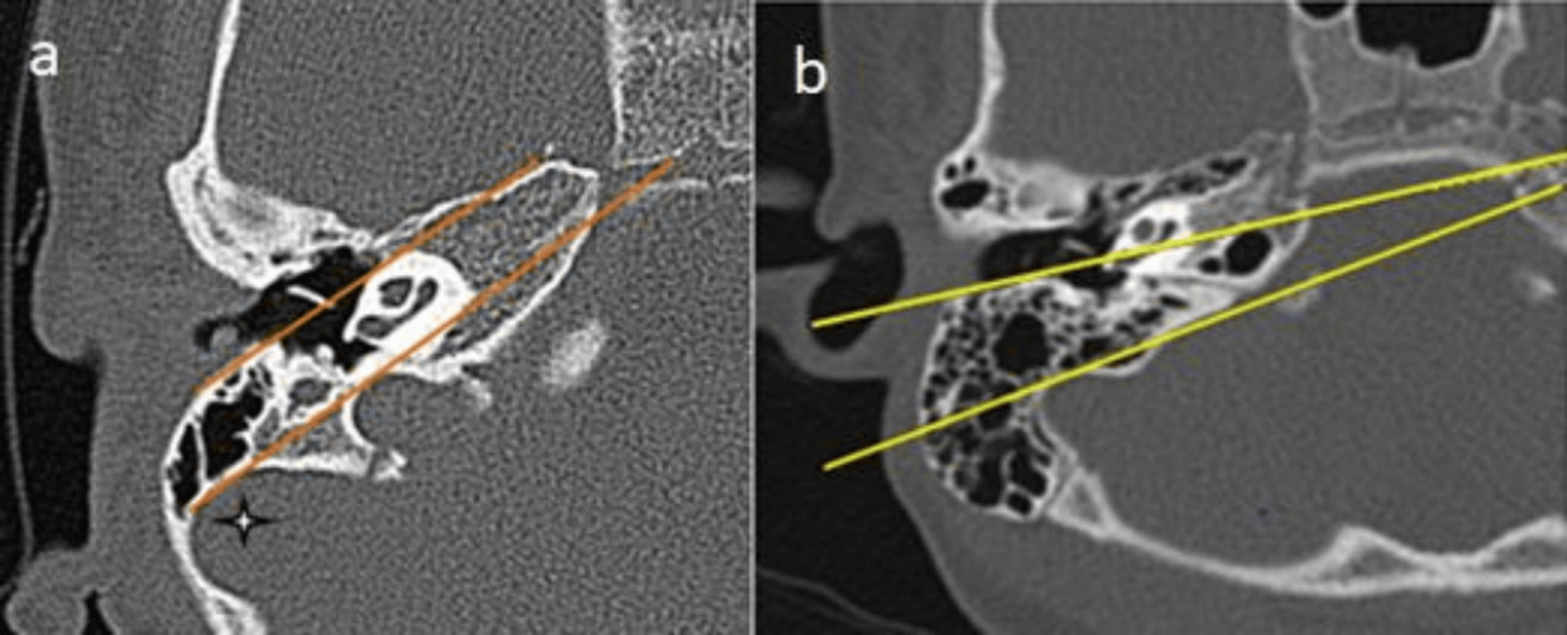
(a,b) Cochlear basal turn in relation to the posterior wall of the external auditory canal and anterior wall of the sigmoid sinus HRCT of the temporal bone showing (a) the cochlear basal turn in between the lines along the posterior wall of the external auditory canal and along the anterior wall of the sigmoid sinus (star) and (b) the cochlear basal turn which is lying outside both lines. HRCT: high-resolution CT

In the study, 77 participants (85.6%) had a favorable cochlear basal turn in relation to the posterior wall of the EAC and the anterior wall of the SS, while 13 participants (14.4%) had an unfavorable configuration. Both favorable and unfavorable types were observed, as shown in Figure 4.

Posterior EAC/long axis of the basal turn line

**Figure 5:**
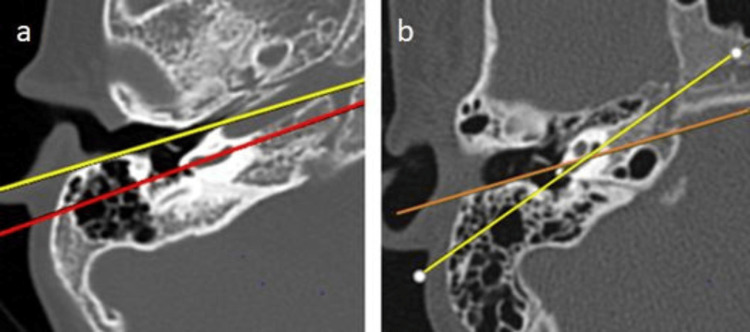
(a,b) Relation between the posterior external auditory canal and the long axis of the basal turn line HRCT of the temporal bone showing that (a) the line along the posterior wall of the external auditory canal (yellow line) and the line along the long axis of the basal turn of the cochlea (red line) are parallel and (b) the line along the posterior wall of the external auditory canal (red line) and the line along the long axis of the basal turn of the cochlea (red line) are intersecting medially. HRCT: high-resolution CT

Among the participants, 65 (72.2%) exhibited a favorable relation between the posterior EAC and the long axis of the basal turn line, while 25 (27.8%) had an unfavorable relation. Both types were observed, as shown in Figure 5.

Lines along the anterior margin of the internal auditory canals (IACs)

Among the participants, 70 (77.8%) had parallel lines along the anterior margin of the IACs, while 20 (22.2%) had angulated and intersecting lines.

Congenital anomalies of the temporal bone

Regarding congenital anomalies of the temporal bone, 87 participants (96.7%) had no anomalies, while three participants (3.3%) presented with isolated large vestibular aqueduct syndrome (LVAS), Mondini malformation, or a bulbous IAC.

Acquired abnormalities of the temporal bone

In terms of acquired abnormalities of the temporal bone, 89 participants (98.9%) exhibited no abnormalities, while one participant (1.1%) was classified as LO Balkany Grade 1.

Total difficulty score

Based on HRCT and MRI data, a 10-point scoring chart was developed, and all patients were given potential difficulty scores (PDS). In each case, surgical times were recorded, and each imaging point on the scoring chart was associated with the surgical times. Eight of the 10 points on the grading system were statistically significant in predicting the surgical procedure's difficulty. We determined that patients with PDS between 0 and 3 (Grade 1) have uneventful and uncomplicated surgery with the shortest intraoperative periods after evaluating the preoperative imaging examinations using a 10-point scoring chart. PDS 4-7 (Grade 2) patients alert the surgeon to considerable surgical complexity and prolonged intraoperative timeframes. A PDS of 8 or above (Grade 3) indicates a lengthy and complicated surgery. The total difficulty scores, derived from various anatomical parameters assessed through HRCT and MRI, were categorized as follows: 29 participants (32.2%) had a score of 0, 27 participants (30.0%) had a score of 1, 21 participants (23.3%) had a score of 2, 11 participants (12.2%) had a score of 3, and two participants (2.2%) had a score of 5, as detailed in Table 2.

**Table 2:** Distribution of participants according to total score The total difficulty score ranges from 1 to 8.

Total difficulty score	Frequency	Percent
0	29	32.2
1.0	27	30.0
2.0	21	23.3
3.0	11	12.2
5.0	2	2.2
Total	90	100.0

Surgical time and grading

The average surgical time recorded was 103.97±25.2 minutes, ranging from 45 to 220 minutes. Surgical procedures were categorized based on duration, as detailed in Table 3: 22 participants (24.4%) fell under Grade 1 (up to 90 minutes), 54 participants (60.0%) were classified as Grade 2 (91-120 minutes), and 14 participants (15.6%) were categorized as Grade 3 (>120 minutes).

**Table 3:** Distribution of participants according to grading based on surgical time

Grading	Frequency	Percent
Grade 1 ( up to 90 min)	22	24.4
Grade 2 (91-120 min)	54	60.0
Grade 3 (>120 min)	14	15.6
Total	90	100.0

Univariate analyses were done initially. Descriptive statistics were used to calculate frequencies of categorical variables, and measures of central tendencies and dispersion were used to describe continuous variables. Bivariate analyses were done using the chi-squared test/Fisher's exact test for qualitative variables. For the quantitative variable, an independent t-test was used. The Pearson correlation coefficient was calculated to find out the correlation between surgical time and different scores. The correlation between imaging and surgical difficulty is shown in Table 4.

**Table 4:** Correlation of surgical time with imaging findings EAC: external auditory canal; SS: sigmoid sinus; IACs: internal auditory canals

Imaging findings	Pearson correlation coefficient	P-value
Degree of mastoid pneumatization	0.120	0.259
Facial recess anatomy	0.198	0.062
Position of jugular bulb	0.178	0.094
Posterior wall of EAC/SS lines	0.064	0.552
Posterior EAC/long axis of the basal turn line	0.134	0.207
Relative position of the basal turn of the cochlea to the MI joint	0.066	0.535
Lines along the anterior margin of IACs	0.091	0.393
Associated congenital anomalies of the temporal bone	0.285	0.006
Associated acquired abnormalities of the temporal bone	0.490	0.0001
Total score	0.469	0.0001

Significant correlations were observed between surgical time and several factors in the study. These included associated congenital anomalies of the temporal bone (p=0.006), associated acquired abnormalities of the temporal bone (p=0.0001), and the total difficulty score (p=0.0001).

These results indicate that specific anatomical features identified in preoperative HRCT and MRI scans can significantly predict the degree of difficulty in cochlear implant surgery, thereby influencing surgical planning and outcomes.

## Discussion

The preoperative assessment of temporal bone anatomy using HRCT and MRI has proven invaluable in predicting the degree of difficulty in cochlear implant surgeries. This study aimed to evaluate these imaging modalities and correlate their findings with intraoperative challenges and surgical duration.

Demographics and surgical distribution

The study's demographic data showed a mean participant age of 7.4 years, predominantly within the 1-5-year age group, consistent with other pediatric cochlear implant studies [8]. The slight male predominance and the higher incidence of right-sided implants align with general clinical practices, where anatomical and procedural considerations often dictate the choice of ear for implantation.

Radiological findings and surgical complexity

The preoperative imaging revealed significant anatomical variations that impacted surgical difficulty. Specifically, 35.6% of participants had hypo-/non-pneumatized mastoids, which can complicate mastoidectomy by reducing the working space and increasing the risk of injury to adjacent structures [5]. Narrow facial recesses and high-riding jugular bulbs, found in 3.3% of participants, respectively, posed additional challenges by limiting visibility and access to critical surgical landmarks such as the round window.

The study identified a statistically significant correlation between preoperative imaging findings and surgical time. The presence of congenital anomalies, like LVAS or Mondini malformation, and acquired abnormalities, such as labyrinthitis ossificans, were associated with increased surgical times (p=0.006 and p=0.0001, respectively). These conditions can complicate electrode insertion and necessitate modifications in the surgical approach, thereby prolonging the procedure [7].

Difficulty score and surgical time

A novel aspect of this study was the development of a total difficulty score based on HRCT and MRI findings. The score effectively predicted surgical complexity, with higher scores correlating with longer surgical times (p=0.0001) [9]. This scoring system provides a practical tool for surgeons to anticipate challenges and plan accordingly, potentially improving surgical outcomes and reducing complications.

The mean surgical time observed in this study was 103.97 minutes, with a range of 45-220 minutes. This duration aligns with existing literature, suggesting that while imaging can highlight potential difficulties, actual surgical time remains influenced by multiple factors, including surgical skill and intraoperative findings.

Comparative analysis with other studies

Comparative analysis with similar studies highlights the consistent role of imaging in surgical planning. For instance, a study by Vaid et al. reported similar correlations between imaging findings and surgical difficulty, reinforcing the reliability of HRCT and MRI in preoperative assessments. Furthermore, studies by Woolley et al. and Antonelli et al. have emphasized the diagnostic yield of HRCT in identifying anatomical variations, underscoring its importance in cochlear implant surgery [10,11].

Limitations and future directions

While the study provides robust evidence supporting the use of HRCT and MRI, it is not without limitations. The sample size, though calculated appropriately, may still limit the generalizability of the findings. Future research could expand the sample size and include multi-center data to validate the difficulty scoring system further [12].

## Conclusions

This study conducted at Saveetha Medical College and Hospital, Chennai, sought to predict the surgical difficulty of cochlear implant procedures using preoperative HRCT and MRI scans. A total of 90 participants were included, with the primary outcome being the correlation between preoperative imaging parameters and surgical time. The findings demonstrated that younger patients, particularly those aged 1-5 years, constituted the majority of the cohort. The study highlighted significant gender distribution and the predominance of right-sided cochlear implants. Key anatomical features, such as mastoid pneumatization and facial recess width, were analyzed, revealing substantial variation among participants.

A notable positive correlation was identified between surgical time and both congenital and acquired anomalies of the temporal bone, supporting the predictive value of HRCT and MRI in assessing surgical complexity. The mean total score derived from imaging assessments proved to be a reliable indicator of intraoperative difficulty. In conclusion, the study validates the utility of HRCT and MRI in preoperative evaluation, offering a viable method to anticipate surgical challenges in cochlear implantation. Further research with larger sample sizes is recommended to consolidate these findings and enhance the predictive accuracy for diverse populations.
